# Efficacy and Safety of Tirzepatide on Weight Loss in Patients Without Diabetes Mellitus: A Systematic Review and Meta‐Analysis of Randomized Controlled Trials

**DOI:** 10.1111/obr.13961

**Published:** 2025-06-13

**Authors:** Sharath Kommu, Param P. Sharma, Rachel M. Gabor

**Affiliations:** ^1^ Department of Hospital Medicine Marshfield Clinic Health System Rice Lake Wisconsin USA; ^2^ Department of Medicine University of Wisconsin School of Medicine and Public Health Madison Wisconsin USA; ^3^ Department of Cardiology Marshfield Clinic Health System Marshfield Wisconsin USA; ^4^ Office of Research Computing and Analytics Marshfield Clinic Research Institute Marshfield Wisconsin USA

**Keywords:** nondiabetics, obesity, overweight, tirzepatide

## Abstract

Tirzepatide has positive effects on weight loss in individuals with overweight or obesity. Considering its broad side‐effect profile, its efficacy and safety in individuals without diabetes mellitus (DM) are yet to be fully understood. To address this, we conducted a comprehensive meta‐analysis of six randomized trials on individuals with overweight or obesity, without DM, which showed that tirzepatide, when compared with placebo, resulted in a change in percentage body weight with a mean difference (MD) of −16.32% (95% CI: −18.35 to −14.29) and change in absolute body weight in kilograms (MD −13.95 kg; −18.83 to −9.07). There were significant reductions in body mass index and waist circumference when compared with placebo, with MDs of −5.89 kg/m^2^ (−8.97 to −2.81) and −12.31 cm (−13.93 to −10.68), respectively. It was associated with gastrointestinal (GI) side effects: nausea (relative risk [RR] 3.11; 2.74–3.54), vomiting (RR 5.94; 4.50–7.85), diarrhea (RR 2.92; 2.53–3.37), and constipation (RR 2.85; 2.38–3.42). Serious adverse events were not statistically significant (RR 0.93; 0.76–1.13), but serious GI events and discontinuation due to adverse events were significant (RRs 3.07; 2.03–4.66, and 2.29; 1.74–3.01, respectively). In conclusion, this meta‐analysis suggests that in patients with overweight or obesity without DM, tirzepatide is effective for significant weight loss. Though the overall risk of serious adverse events is not higher compared with placebo, it carries an elevated risk of GI side effects, serious GI events, and discontinuation due to adverse effects.

**Abbreviations**
AHI: apnea–hypopnea index
BMI: body mass index
CI: confidence interval
DM: diabetes mellitus
FDA: Food and Drug Administration
GI: gastrointestinal
GIP: glucose‐dependent insulinotropic polypeptide
GLP‐1: glucagon‐like peptide‐1
GLP‐1 RA: glucagon‐like peptide‐1 receptor agonist
HbA1C: glycated hemoglobin
HDL: high‐density lipoprotein
LDL: low‐density lipoprotein
MD: mean difference
MTD: maximum tolerated dose
OR: odds ratio
OSA: obstructive sleep apnea
PAP: positive airway pressure
PRISMA: Preferred Reporting Items for Systematic Reviews and Meta‐Analyses
RCT: randomized controlled trial
RoB 2: version 2 of the Cochrane risk‐of‐bias tool for randomized trials
RR: relative risk
VLDL: very‐low‐density lipoprotein

## 1 Introduction

Obesity affects approximately 650 million adults globally and is linked to numerous complications, including type 2 diabetes mellitus (DM), sleep apnea, and cardiovascular diseases [1, 2]. For many years, lifestyle‐based interventions have been the cornerstone of obesity treatment. However, maintaining weight loss through lifestyle changes and caloric restriction alone has proven difficult for many individuals. With the advent of new medications showing promising results, significant progress has been made in obesity management. Notably, the glucagon‐like peptide‐1 (GLP‐1) receptors, as targets for weight loss, have garnered significance. GLP‐1 receptor agonists (GLP‐1 RAs), such as semaglutide, which target pathways of endogenous nutrient‐stimulated hormones, have revolutionized the management of obesity in recent times [3].

In addition to GLP‐1, researchers identified another nutrient‐stimulated hormone, glucose‐dependent insulinotropic polypeptide (GIP), as a promising therapeutic target [4, 5]. Targeting both GIP and GLP‐1 is believed to enhance weight loss compared with medications that focus solely on GLP‐1. Tirzepatide, a dual receptor agonist that activates both GLP‐1 and GIP receptors, has demonstrated favorable weight loss outcomes in several clinical trials. As a result, tirzepatide received FDA approval for chronic weight management [6].

The SURMOUNT trials represent the primary investigations into tirzepatide's role in weight loss, showing promising results in individuals both with and without DM [7, 8, 9, 10]. However, like GLP‐1 RAs, tirzepatide can cause a range of side effects, particularly GI, which may limit its use. Given tirzepatide's dual action on GLP‐1 and GIP receptors, it could be hypothesized that, while it offers greater weight loss benefits compared with GLP‐1 RAs, it may also present a higher side effect burden due to its additional impact on GIP.

Although there are several meta‐analyses exploring the efficacy and safety of tirzepatide for weight loss, these often include patients with and without DM [11, 12, 13, 14]. Meta‐analyses that focus exclusively on the effects of tirzepatide in individuals without DM are limited. To date, there is only one such meta‐analysis, but it includes data from only three trials, which restricts its conclusions [15]. A comprehensive and up‐to‐date meta‐analysis incorporating all relevant trials in individuals without DM is needed to provide a more detailed understanding of tirzepatide's effects, especially considering its extensive side‐effect profile. Our study aims to address this gap and provide a clearer, more nuanced understanding of the benefits and risks of tirzepatide in individuals without DM.

## 2 Methods

The Preferred Reporting Items for Systematic Reviews and Meta‐Analyses (PRISMA) guidelines were followed to identify studies eligible for inclusion in this meta‐analysis. The study is registered in PROSPERO with the ID CRD42024564649.

We conducted a comprehensive search using the terms “Tirzepatide,” “weight loss,” “overweight,” and “obesity.” Searches were performed on MEDLINE (via PubMed) and ClinicalTrials.gov, covering the period from July 1, 2019, to June 30, 2024. Detailed search strategies are provided in the Supporting Information (Table S1).

To be included, studies had to meet the following criteria: (1) randomized, placebo‐controlled trials of tirzepatide treatment, (2) studies involving patients with overweight or obesity, and (3) studies involving human adults without a diagnosis of DM.

We thoroughly reviewed the search results for potential inclusion and manually checked the references of identified studies to uncover any additional relevant trials. Two authors (SK and PS) were responsible for the study selection. One author (SK) screened the records, while the other (PS) cross‐checked them. Any disagreements were resolved through discussion between the authors.

### 2.1 Data Extraction

After identifying the relevant studies, we extracted the data needed for our meta‐analysis. This included the study name, year of publication, study population, the dosage of tirzepatide, study duration, various weight loss efficacy outcomes, and reported side effects or adverse events. Only efficacy outcomes and side effects that were common across most trials were included in the analysis. Two authors, SK and PS, were responsible for data extraction: SK extracted the data, while PS verified it. Any disagreements were resolved through discussion, with input from a third author, RG, if necessary.

The efficacy outcomes extracted for this meta‐analysis included the following: percentage change in weight, change in weight (kg), percentage of participants achieving body weight reductions of > 5%, > 10%, > 15%, > 20%, and > 25%, changes in body mass index (BMI), waist circumference (cm), glycated hemoglobin (HbA1C), fasting glucose (mg/dL), systolic/diastolic blood pressure (mm Hg), and lipid panel results (total cholesterol, low‐density lipoprotein [LDL], high‐density lipoprotein [HDL], triglycerides, very‐low‐density lipoprotein [VLDL], and free fatty acids).

Data on common side effects were also extracted, including nausea, vomiting, diarrhea, constipation, dyspepsia, eructation, cholelithiasis, gallbladder disorders, pancreatitis, hypoglycemia, injection site reactions, alopecia, the percentage of participants experiencing serious adverse events, serious gastrointestinal (GI) events, death, and discontinuation due to adverse events.

### 2.2 Quality Assessment

All included studies were independently evaluated for quality using the Cochrane Risk of Bias tool for randomized trials (ROB2) [16]. This tool assesses key study characteristics, including the randomization process, deviations from intended interventions, missing outcome data, measurement of outcomes, and the selection of reported results. Each indicator was classified as having a low, high, or unclear risk of bias. Studies were considered low risk if all indicators were deemed low risk. If any indicator was found to have a high risk, the study was classified as high risk. In cases where the risk was unclear for one or more indicators, the study was categorized as unclear risk. Two authors, SK and PS, conducted the quality assessments, resolving disagreements through discussion. A third author (RG) was consulted to reach a consensus if necessary.

### 2.3 Statistical Analysis

Once the studies were identified, data synthesis was conducted for the various efficacy and safety parameters. We utilized the meta package in R software (version 4.3.14) with the inverse variance method to analyze data related to various weight loss parameters and adverse events [17]. For continuous data, such as percentage change in weight, we expressed the effect size as mean difference (MD) with 95% confidence intervals (CIs). For dichotomous data, we used relative risk or risk ratios (RR) or odds ratios (OR), along with 95% CIs.

To assess heterogeneity and determine the need for a random effects model, we calculated two measures: Higgins and Thompson's *I* ^2^ statistic and the heterogeneity variance (*τ* ^2^). If the *p* value for heterogeneity was less than 0.1, a random effects model was deemed necessary. A calculated *p* value of less than 0.05 was considered significant evidence of a treatment difference.

## 3 Results

**FIGURE 1:**
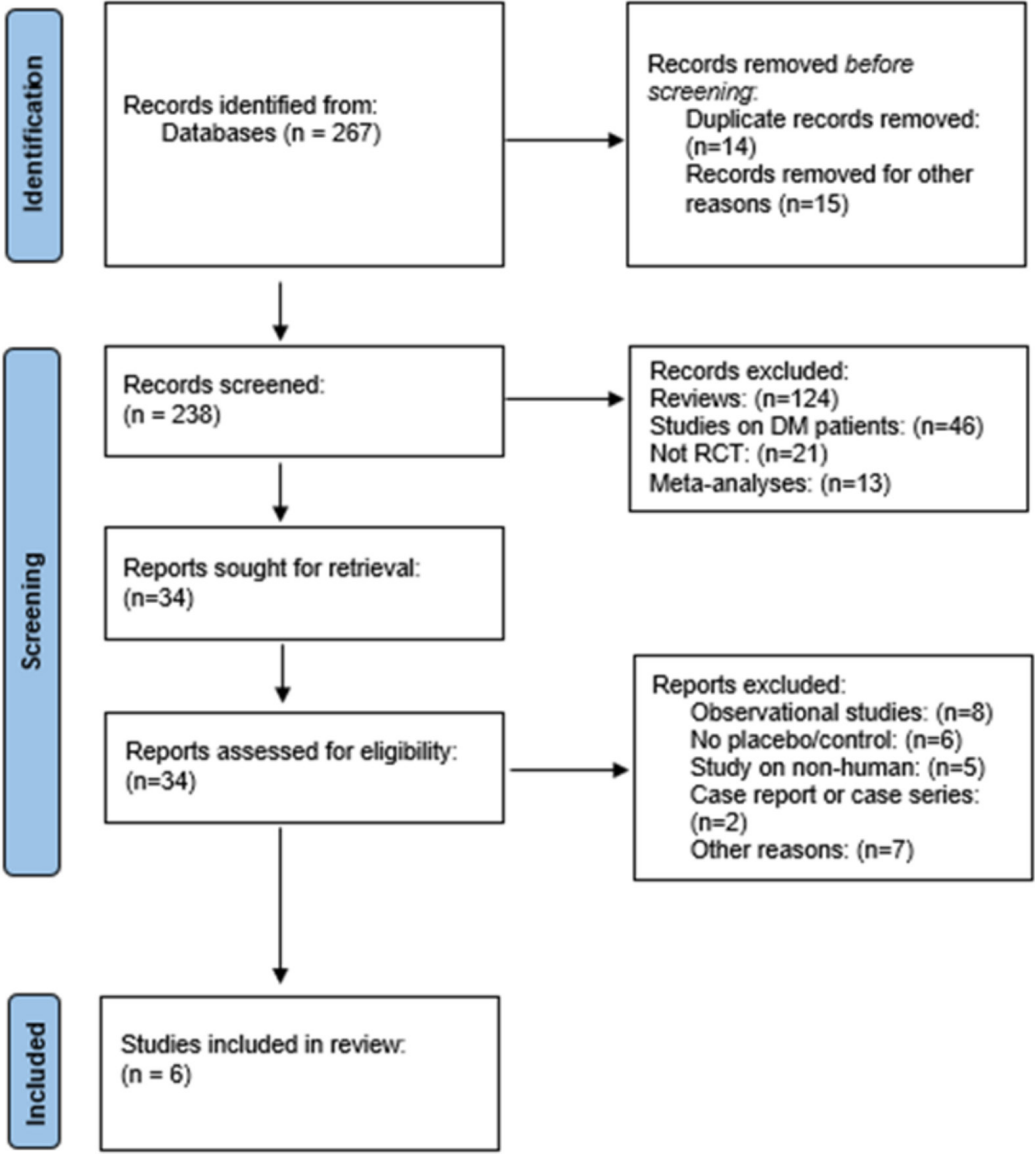
PRISMA algorithm showing the selection of studies for the meta‐analysis. PRISMA—Preferred Reporting Items for Systematic Review and Meta‐Analysis. DM—diabetes mellitus, RCT—randomized controlled trial.

The online data search yielded 267 references. Following the PRISMA guidelines and applying our inclusion criteria, we identified six studies eligible for inclusion in the meta‐analysis (Figure 1).

### 3.1 Characteristics of Studies

Table 1 presents the six studies included in this meta‐analysis and their characteristics. All studies involved participants without DM. Most of the included studies belong to the SURMOUNT trial group except for one (NCT04081337, as documented on ClinicalTrials.gov) [7, 9, 10, 18, 19, 20]. The SURMOUNT trials included here are phase 3 randomized controlled trials (RCTs), spanning between 52 and 88 weeks, whereas the NCT04081337 study is a shorter phase 1 trial lasting 18 weeks. Notably, the SURMOUNT‐CN trial was conducted exclusively in China [18].

**TABLE 1:** Characteristics of the studies included in the meta‐analysis.

Study name	Type of study	Duration	Assignment and dosage	Study population	Study location	Tirzepatide (*n*)	Placebo (*n*)
Jastreboff et al. SURMOUNT 1 [7]	Phase 3 multicenter, double‐blind, randomized, placebo‐controlled trial	72 weeks	Participants were randomly assigned in a 1:1:1:1 ratio to receive tirzepatide 5, 10, or 15 mg, or placebo, subcutaneously, once weekly	≥ 18 years, with BMI ≥ 30, or a BMI ≥ 27 with at least one weight‐related complication and who reported ≥ 1 unsuccessful dietary effort to lose weight	119 sites in nine countries (Argentina, Brazil, China, India, Japan, Mexico, Russian Federation, Taiwan, United States)	5 mg (*n* = 630) 10 mg (*n* = 636) 15 mg (*n* = 630)	643
Wadden et al. SURMOUNT 3 [9]	Phase 3 multicenter, randomized, parallel‐arm, double‐blind, placebo‐controlled trial	84 weeks	Participants were randomly assigned in a 1:1 ratio the MTD of tirzepatide (10 or 15 mg) or placebo subcutaneously, once weekly	≥ 18 years, with BMI ≥ 30, or a BMI ≥ 27 with at least one weight‐related complication and who reported ≥ 1 unsuccessful dietary effort to lose weight	62 medical research centers in the United States, Argentina, and Brazil	287	292
Aronne et al. SURMOUNT 4 [10]	Phase 3 randomized withdrawal study with a 36‐week, open‐label tirzepatide lead‐in period followed by a 52‐week, double‐blind, placebo‐controlled period	88 weeks	At the end of the lead‐in period, participants were randomly assigned in a 1:1 ratio the MTD of subcutaneous once weekly tirzepatide (10 or 15 mg) or placebo.	≥ 18 years, with BMI ≥ 30, or a BMI ≥ 27 with at least one weight‐related complication and who reported ≥ 1 unsuccessful dietary effort to lose weight	70 sites in Argentina, Brazil, Taiwan, and the United States	335	335
Zhao et al. SURMOUNT CN [18]	Phase 3 multicenter, randomized, double‐blind, placebo‐controlled trial	52 weeks	Participants were randomly assigned in a 1:1:1 ratio to receive subcutaneous once weekly tirzepatide 10 mg, tirzepatide 15 mg, or placebo	≥ 18 years, with BMI ≥ 28, or a BMI ≥ 24 with at least one weight‐related complication and who reported ≥ 1 unsuccessful dietary effort to lose weight	29 centers in China	10 mg (*n* = 70) 15 mg (*n* = 71)	69
Malhotra et al. SURMOUNT‐OSA [19] Trial 1: participants who were unable or unwilling to use PAP therapy Trial 2: participants who had been using PAP therapy for ≥ 3 consecutive months at the time of screening and who planned to continue PAP therapy during the trial	Two phase 3, multicenter, parallel‐group, double‐blind, randomized, controlled trials (Trial 1 and Trial 2)	52 weeks	Participants were randomly assigned to Trial 1 or Trial 2 and randomly assigned in a 1:1 ratio to tirzepatide or placebo subcutaneously once weekly. Dose was gradually escalated to MTD of 10 or 15 mg	≥ 18 years, with a diagnosis of moderate‐to‐severe OSA (AHI ≥ 15 events/h) and BMI ≥ 30 (≥ 27 in Japan)	60 sites across nine countries (Australia, Brazil, China, Czech Republic, Germany, Japan, Mexico, Taiwan, and the United States)	Trial 1 (*n* = 114) Trial 2 (*n* = 120)	Trial 1 (*n* = 120) Trial 2 (*n* = 115)
ClinicalTrials.gov ID NCT04081337 [20]	Phase 1 randomized, placebo‐controlled, parallel‐arm study	18 weeks	Randomly assigned to receive tirzepatide 15 mg or placebo, once‐weekly subcutaneously	18–60 years, with a BMI of 30–45, with a stable weight for 1 month prior to the time of screening	United States	27	28

Abbreviations: AHI—apnea–hypopnea index; BMI—body mass index; MTD—maximum tolerated dose; *n*—number of patients; OSA—obstructive sleep apnea; PAP—positive airway pressure.

In these studies, overweight is defined as a BMI of ≥ 27, except for SURMOUNT‐CN, where the threshold for overweight is a BMI of ≥ 24 [18]. Similarly, obesity is defined as a BMI of ≥ 30 in most trials, except for SURMOUNT‐CN, where obesity is defined as a BMI of ≥ 28, and in the Japanese cohort of the SURMOUNT‐OSA trial, where a BMI of ≥ 27 defines obesity [18, 19]. These lower thresholds reflect the definitions of obesity in China and Japan, where health‐related problems tend to arise at lower BMIs compared with Western populations [21, 22, 23].

While all studies included participants with overweight or obesity, the SURMOUNT‐OSA trial specifically targeted patients with obesity and moderate‐to‐severe obstructive sleep apnea (OSA), characterized by an apnea‐hypopnea index (AHI) of ≥ 15 events per hour [19]. This trial comprised two segments: Trial 1, which included participants who were either unable or unwilling to use positive airway pressure (PAP) therapy, and Trial 2, which involved participants who had been using PAP therapy for three or more consecutive months at the time of screening and intended to continue this therapy throughout the trial [19].

### 3.2 Risk of Bias Assessment

**FIGURE 2:**
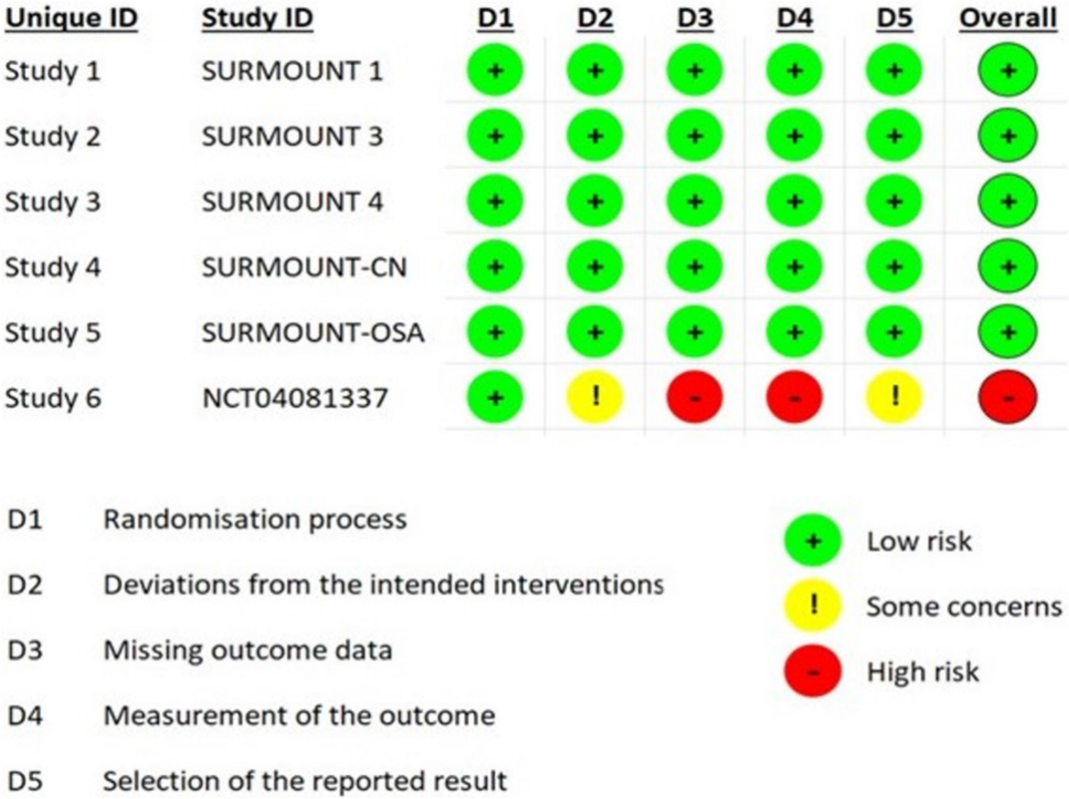
Risk of bias for the six studies assessed using version 2 of the Cochrane risk‐of‐bias tool (ROB2).

We assessed various parameters for evaluating the risk of bias, including the randomization process, deviations from intended interventions, missing outcome data, outcome measurement, and selection of reported results, using the revised Cochrane Risk‐of‐Bias tool for randomized trials (RoB 2), as illustrated in Figure 2. No major bias was identified in five of the six studies included in this analysis. However, the study NCT04081337 exhibited a potentially high risk of bias due to the lack of detailed information available regarding its methodology.

### 3.3 Main Analysis

Each outcome and side effect were analyzed and synthesized using forest plots. Heterogeneity was observed in all efficacy parameters except for the change in HbA1C (%), change in blood glucose (mg/dL), change in HDL‐C (%), and change in free fatty acids (%). No heterogeneity was found among the safety parameters. The specific reasons for the heterogeneity in most efficacy parameters remain unclear but are likely related to variations in dosages and follow‐up durations across the studies. When heterogeneity was present, a random effects model was employed for the analysis.

### 3.4 Efficacy Parameters

**FIGURE 3:**
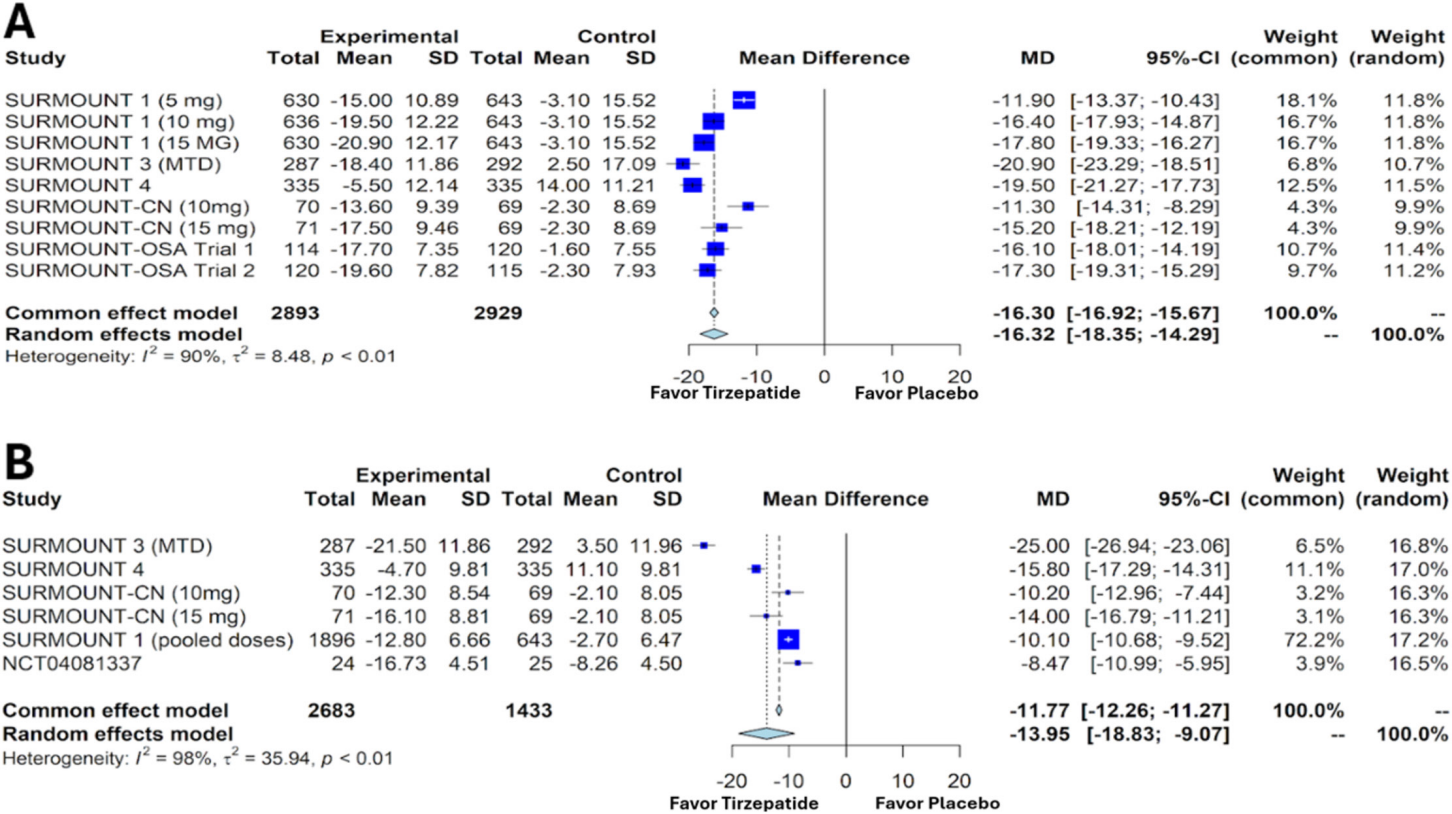
Forest plots showing the change in body weight in percentage (A) and kilograms (B) in patients with overweight or obesity without diabetes mellitus treated with tirzepatide versus placebo. SD—standard deviation, MD—mean difference, CI—confidence interval, MTD—maximum tolerated dose.

Our meta‐analysis revealed significant improvements in various weight‐related parameters with tirzepatide. Compared with placebo, once‐weekly tirzepatide led to a substantial decrease in the percentage change in body weight, with an MD of −16.32% and a 95% CI of −18.35 to −14.29 (*p* < 0.0001) (Figure 3A). Similarly, the reduction in body weight measured in kilograms was also significant, showing an MD of −13.95 kg and a 95% CI of −18.83 to −9.07 (*p* < 0.0001) (Figure 3B).

**FIGURE 4:**
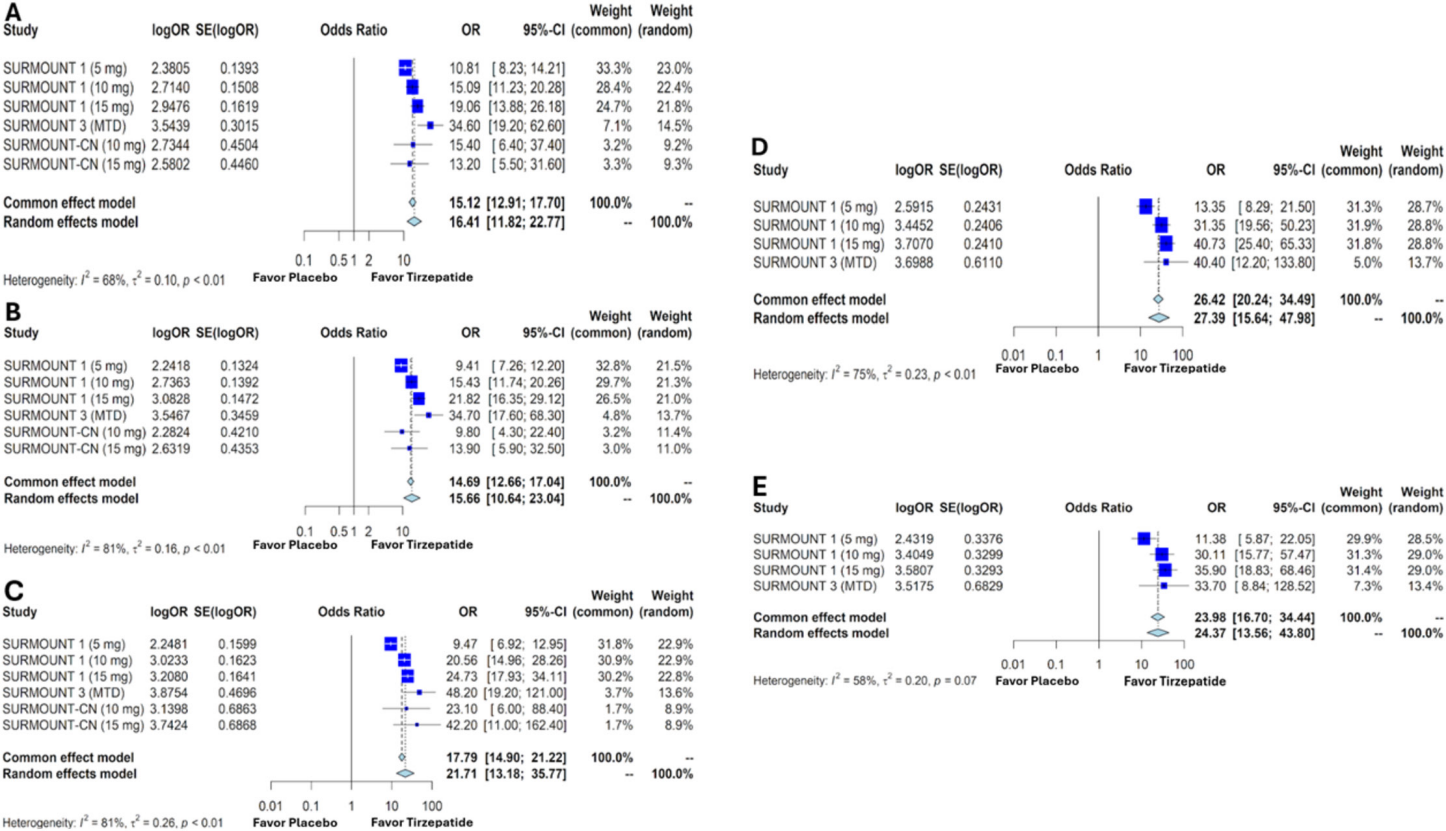
Forest plots showing the odds ratios of weight loss of ≥ 5% (A), ≥ 10% (B), ≥ 15% (C), ≥ 20% (D), and ≥ 25% (E) in patients with overweight or obesity without diabetes mellitus treated with tirzepatide versus placebo. OR—odds ratio, SE—standard error, CI—confidence interval, MTD—maximum tolerated dose.

The pooled OR for achieving weight reductions of at least 5%, 10%, 15%, 20%, and 25% with once‐weekly tirzepatide compared with placebo was as follows:

- For a 5% reduction: OR 16.41 (95% CI: 11.82–22.77, *p* < 0.0001) (Figure 4A)
- For a 10% reduction: OR 15.66 (95% CI: 10.64–23.04, *p* < 0.0001) (Figure 4B)
- For a 15% reduction: OR 21.71 (95% CI: 13.18–35.77, *p* < 0.0001) (Figure 4C)
- For a 20% reduction: OR 27.39 (95% CI: 15.64–47.98, *p* < 0.0001) (Figure 4D)
- For a 25% reduction: OR 24.37 (95% CI: 13.56–43.80, *p* < 0.0001) (Figure 4E)

Additionally, once‐weekly tirzepatide resulted in decreases in BMI and waist circumference, with MDs of −5.89 kg/m^2^ (95% CI: −8.97 to −2.81, *p* = 0.0002) (Figure S1A) and −12.31 cm (95% CI: −13.93 to −10.68, *p* < 0.0001) (Figure S1B), respectively.

These findings underscore the significant beneficial effects of tirzepatide on weight‐related parameters.

Our study also demonstrated improvements in other parameters with once‐weekly tirzepatide compared with placebo. Notably, there was a modest reduction in HbA1C (%), with a change of −0.41 (95% CI: −0.50 to −0.33, *p* < 0.0001) (Figure S2A). Additionally, fasting glucose (mg/dL) showed a change of −10.09 (95% CI: −11.81 to −8.38, *p* < 0.0001) (Figure S2B). We observed reductions in both systolic and diastolic blood pressures, with changes of −6.63 mmHg (95% CI: −8.41 to −4.85, *p* < 0.0001) (Figure S3A) and −4.02 mmHg (95% CI: −6.20 to −1.85, *p* = 0.0003) (Figure S3B), respectively. These improvements suggest that tirzepatide may provide additional benefits beyond weight loss.

Additionally, we analyzed changes in the lipid profile, comparing once‐weekly subcutaneous tirzepatide to placebo, as shown in Figures S4 and S5. Tirzepatide demonstrated favorable effects on the lipid panel. The MD in the percentage change compared with placebo was −5.57 (95% CI: −9.39 to −1.75, *p* = 0.0042) for total cholesterol (Figure S4A), −24.48 (95% CI: −32.36 to −16.59, *p* < 0.0001) for triglycerides (Figure S4B), −6.61 (95% CI: −12.34 to −0.88, *p* = 0.024) for LDL cholesterol (Figure S4C), −24.52 (95% CI: −31.73 to −17.31, *p* < 0.0001) for VLDL (Figure S5A), −17.35 (95% CI: −22.17 to −12.54, *p* < 0.0001) for free fatty acids (Figure S5B), and 9.80 (95% CI: 7.88–11.71, *p* < 0.0001) for HDL cholesterol (Figure S5C), all with statistically significant *p* values.

### 3.5 Safety Parameters

**FIGURE 5:**
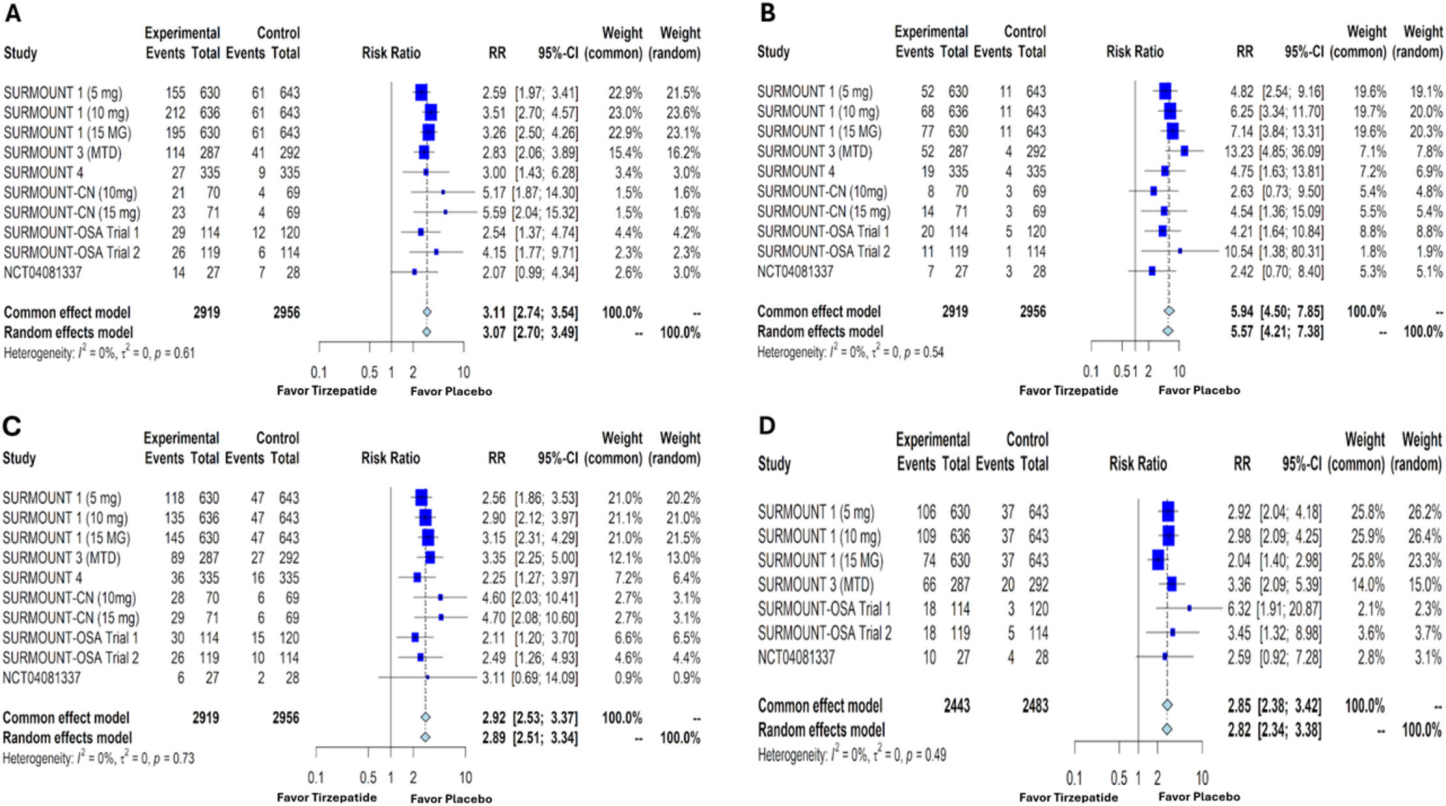
Forest plots showing the gastrointestinal side effects—nausea (A), vomiting (B), diarrhea (C), and constipation (D) in patients with overweight or obesity without diabetes mellitus treated with tirzepatide versus placebo. RR—relative risk, CI—confidence interval, MTD—maximum tolerated dose.

Although tirzepatide demonstrated significant improvements in weight loss parameters, GI side effects were a major limiting factor across most studies. Our meta‐analysis revealed that compared with placebo, the incidence of nausea and vomiting with once‐weekly tirzepatide was significantly higher, with RR of 3.11 (95% CI: 2.74–3.54, *p* < 0.0001) (Figure 5A) and 5.94 (95% CI: 4.50–7.85, *p* < 0.0001) (Figure 5B), respectively. Similarly, the RRs for diarrhea and constipation were 2.92 (95% CI: 2.53–3.37, *p* < 0.0001) (Figure 5C) and 2.85 (95% CI: 2.38–3.42, *p* < 0.0001) (Figure 5D), both statistically significant. Dyspepsia and eructation were also more common with tirzepatide, with RRs of 2.59 (95% CI: 2.05–3.27, *p* < 0.0001) (Figure S6A) and 8.05 (95% CI: 4.80–13.50, *p* < 0.0001) (Figure S6B), respectively.

The risks of gallbladder disorder, cholelithiasis, and pancreatitis were not significantly different between tirzepatide and placebo, with RRs of 1.26 (95% CI: 0.70–2.27, *p* = 0.43) (Figure S7A), 1.18 (95% CI: 0.69–2.03, *p* = 0.55) (Figure S7B), and 1.51 (95% CI: 0.42–5.38, *p* = 0.52) (Figure S7C), respectively.

Non‐GI side effects, such as hypoglycemia, injection site reactions, and alopecia, were more frequent with tirzepatide compared with placebo, with RRs of 6.26 (95% CI: 2.44–16.01, *p* < 0.0001) (Figure S8A), 14.17 (95% CI: 7.48–26.85, *p* < 0.0001) (Figure S8B), and 5.50 (95% CI: 3.51–8.64, *p* < 0.0001) (S8C), respectively.

**FIGURE 6:**
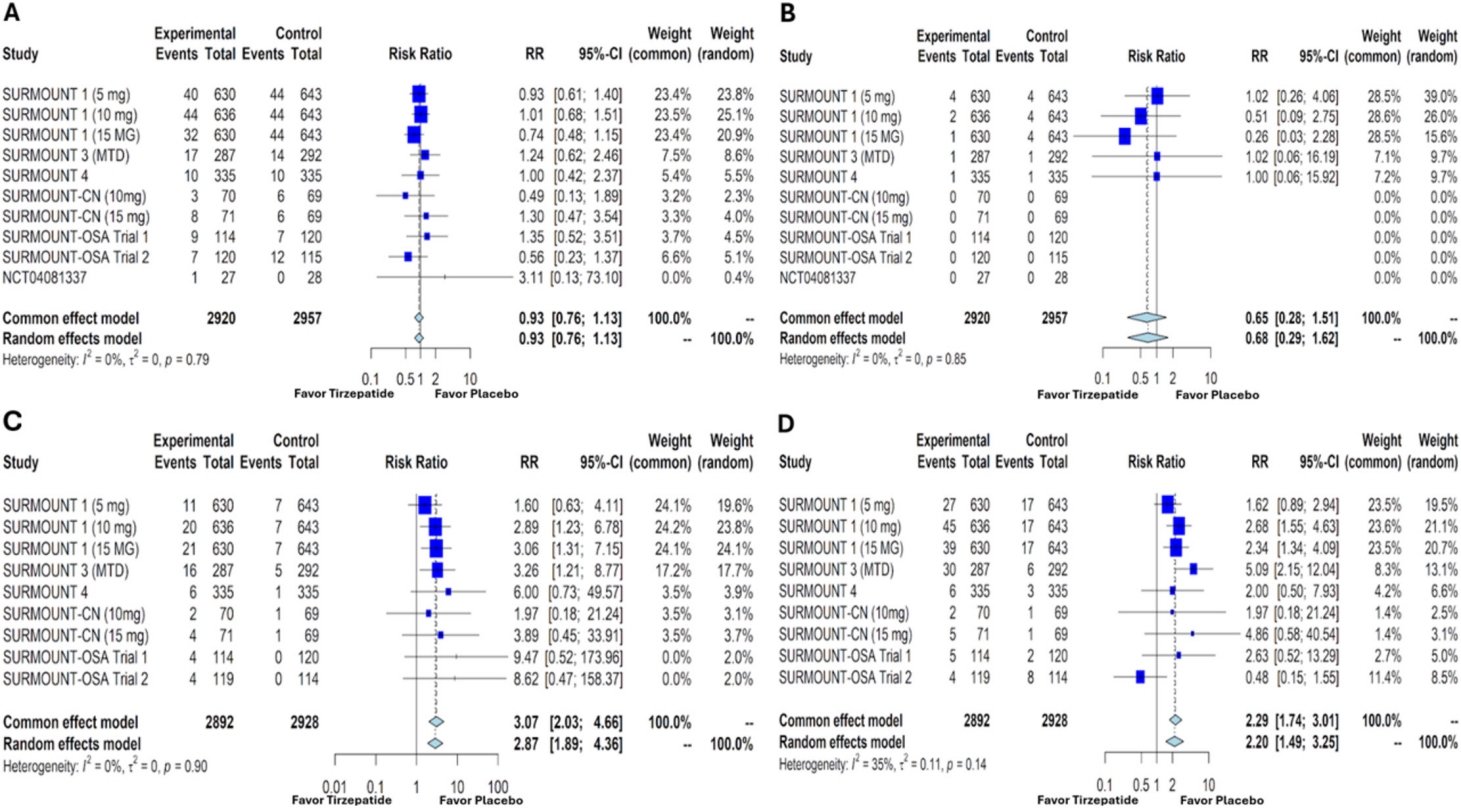
Forest plots showing the participants with serious adverse events(A), death (B), serious gastrointestinal events (C), and participants discontinuing the medication due to adverse events (D) in patients with overweight or obesity without diabetes mellitus treated with tirzepatide versus placebo. RR—relative risk, CI—confidence interval, MTD—maximum tolerated dose.

Despite the side effects associated with tirzepatide, the RR for serious adverse events with once‐weekly tirzepatide compared with placebo was 0.93 (95% CI: 0.76–1.13, *p* = 0.45), which was not statistically significant (Figure 6A). Similarly, the RR for mortality showed no significant difference between tirzepatide and placebo (RR: 0.65, 95% CI: 0.28–1.51, *p* = 0.32) (Figure 6B). However, the RR for serious GI events was statistically significant at 3.07 (95% CI: 2.03–4.66, *p* < 0.0001) (Figure 6C), and the percentage of participants discontinuing tirzepatide due to adverse events was also significantly higher, with an RR of 2.29 (95% CI: 1.74–3.01, *p* < 0.0001) (Figure 6D).

## 4 Discussion

Tirzepatide stands out among new weight loss medications for its dual action targeting GLP‐1 and GIP receptors. Like GLP‐1 RAs, tirzepatide aids weight loss by increasing insulin secretion, slowing gastric emptying, and suppressing appetite [24]. Preclinical studies also show that GIP receptor activation lowers body weight by reducing food intake [25]. Additionally, tirzepatide's GIP agonism enhances its insulinotropic effect, further improving blood glucose control. Finan et al. first described the synergistic action of GIP and GLP‐1, introducing the term “twincretin” to capture this dual mechanism [26].

The SURPASS trials highlighted tirzepatide's effectiveness in lowering blood sugar levels in patients with type 2 DM and showcased its significant weight loss benefits [27, 28, 29, 30, 31, 32]. The SURMOUNT trials further confirmed tirzepatide's weight loss effects, leading to its FDA approval as a treatment for weight management [7, 8, 9, 10]. While previous meta‐analyses have explored tirzepatide's impact on weight loss in individuals with overweight or obesity, our meta‐analysis offers distinct advantages. First, we focused exclusively on patients without DM, resulting in a more homogeneous study population. To our knowledge, only one previous meta‐analysis included patients without DM, but it was limited to just three studies [15]. In contrast, our analysis includes six studies, providing more current and comprehensive data. Second, we conducted an in‐depth evaluation of tirzepatide's efficacy, carefully examining key limitations and side effects that could influence its clinical use. This rigorous assessment positions our meta‐analysis as a valuable and up‐to‐date resource on tirzepatide's role in weight management for individuals without DM.

For the outcome of weight loss (%), the meta‐analysis by Pan et al., which included studies with patients with and without DM, demonstrated that tirzepatide 15 mg once‐weekly led to significant weight loss compared with placebo, with a MD of −14.80% (95% CI: −17.39 to −12.20) [12]. Similarly, Liu et al.'s study in individuals without DM found that tirzepatide resulted in a weight reduction of −18.7% (95% CI: −21.3% to −16.2%) compared with placebo [15]. In comparison, our meta‐analysis, which is also in individuals without DM, showed a weight reduction of −16.32% (95% CI: −18.35 to −14.29) relative to placebo (Figure 3A). Furthermore, de Mesquita et al. reported that in a mixed cohort (with and without DM), tirzepatide led to an absolute weight loss of −7.7 kg (95% CI: −11.0 to −4.4) versus placebo [14]. In contrast, our study, focusing solely on patients without DM, observed a weight loss of −13.95 kg (95% CI: −18.83 to −9.07) compared with placebo (Figure 3B).

The study by Liu et al. involving patients without DM reported the OR for weight reduction of 5%, 10%, 15%, 20%, and 25% with tirzepatide compared with placebo as follows: 21.14 (95% CI: 12.64–35.35), 20.03 (95% CI: 12.47–32.17), 23.61 (95% CI: 13.46–41.41), 26.52 (95% CI: 14.69–47.88), and 26.67 (95% CI: 18.25–38.96) [15]. In comparison, our study reported these values as 16.41 (95% CI: 11.82–22.77), 15.66 (95% CI: 10.64–23.04), 21.71 (95% CI: 13.16–35.77), 27.39 (95% CI: 15.64–47.98), and 24.37 (95% CI: 13.56–43.80), respectively (Figure 4). Although our values are not identical to the results in Liu et al.'s study, they are comparable and statistically significant in both studies. In both studies, the OR tends to increase with higher percentages of body weight reduction, underscoring tirzepatide's effectiveness for greater weight loss.

When comparing tirzepatide to semaglutide, a meta‐analysis involving patients with overweight or obesity without DM found that the percentage weight loss with semaglutide compared with placebo had an MD of −11.49% (95% CI: −13.12 to −9.86) [33]. In contrast, our study on tirzepatide, which also focused on patients without DM, reported a greater percentage of weight loss with an MD of −16.32% (95% CI: −18.35 to −14.29) (Figure 3A). Additionally, the absolute weight loss from the same study on semaglutide indicated an MD of −11.74 kg (95% CI: −13.53 to −9.94) [33]. In comparison, our analysis of tirzepatide revealed an MD of −13.95 kg (95% CI: −18.83 to −9.07) (Figure 3B). Similarly, a real‐world observational study on tirzepatide versus semaglutide revealed the mean on‐treatment change in body weight was −15.3% (95% CI, −16.0% to −14.5%) for tirzepatide versus −8.3% (95% CI, −9% to −7.6%) for semaglutide at 12 months [34]. This greater weight loss with tirzepatide relative to semaglutide is likely attributed to tirzepatide's dual receptor agonistic effects.

In addition to promoting weight loss, tirzepatide also led to a reduction in HbA1C levels. Our pooled analysis indicated that tirzepatide resulted in a change in HbA1C of −0.41 compared with placebo, with a 95% CI of −0.50 to −0.33 (Figure S2A). Similarly, Liu et al. found that tirzepatide produced a change in HbA1C of −0.42 (95% CI: −0.58 to −0.26) when compared with placebo [15]. These reductions were lower than those observed in patients with type 2 DM, as demonstrated in the SURPASS‐1 trial, where the treatment difference in HbA1C compared with placebo ranged from −1.91 to −2.11 across various doses of tirzepatide [27]. The lesser decrease in HbA1C among patients without DM2 compared with those with DM2 may be attributed to tirzepatide's mechanism of action, which involves glucose‐dependent insulin secretion, resulting in a more pronounced glucose‐lowering effect when blood glucose levels are elevated.

Our pooled analyses revealed that tirzepatide also led to a significant decrease in systolic and diastolic blood pressures (Figure S3). Additionally, a favorable response was observed in various lipid parameters (Figures S4 and S5). Although the exact mechanisms behind these effects remain unclear, the GIP agonistic action of tirzepatide may play a role in its lipid‐lowering properties. These effects could potentially provide additional cardiovascular benefits associated with tirzepatide.

**FIGURE 7:**
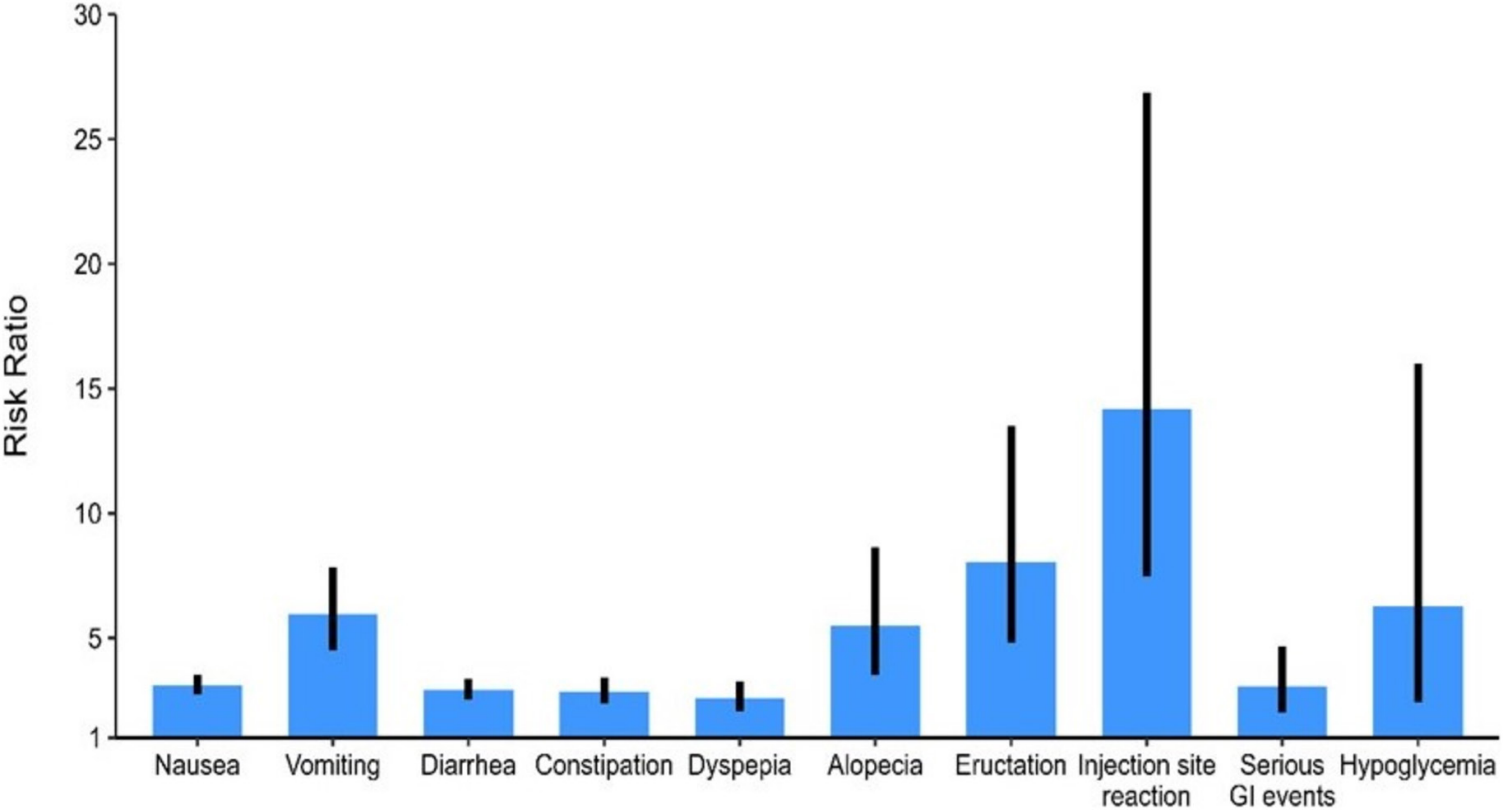
Graph showing the relative risks (depicted as the blue bars) and the corresponding 95% confidence intervals (black lines) of the side effects in patients with overweight or obesity without diabetes mellitus treated with tirzepatide versus placebo. GI—gastrointestinal.

It is important to note that, despite the numerous beneficial effects of tirzepatide, various RCTs and meta‐analyses have shown an increased risk of side effects, regardless of the presence of DM. Our study specifically highlights these side effects in individuals with overweight or obesity without DM, which may ultimately limit the drug's utility. The most prominent among these are GI side effects, including nausea, vomiting, eructation, dyspepsia, diarrhea, and constipation. Figure 7 summarizes the prominent side effects of tirzepatide in patients without DM, as analyzed in our study.

Given tirzepatide's dual receptor agonistic effects, one might expect it to cause more side effects compared with GLP‐1 RAs like semaglutide. While our study is not a direct comparison between semaglutide and tirzepatide, we can make some inferences from a similar study that investigated semaglutide versus placebo in a comparable population (individuals with overweight or obesity without DM) [33]. That study reported higher odds of nausea, vomiting, diarrhea, and constipation with semaglutide versus placebo, with ORs of 4.06 (95% CI: 3.43–4.81), 4.43 (95% CI: 3.48–5.63), 2.10 (95% CI: 1.77–2.49), and 2.43 (95% CI: 2.01–2.94), respectively [33]. In our study, we used RR to estimate the treatment differences for these GI outcomes. The RRs for tirzepatide versus placebo were 3.11 (95% CI: 2.74–3.54) for nausea, 5.94 (95% CI: 4.50–7.85) for vomiting, 2.92 (95% CI: 2.53–3.37) for diarrhea, and 2.85 (95% CI: 2.38–3.42) for constipation (Figure 5). These results suggest that the risk of GI side effects is fairly comparable between tirzepatide and semaglutide.

For the other GI side effects, the same meta‐analysis on semaglutide versus placebo reported a higher incidence of gallbladder disorders (OR 1.26, 95% CI: 1.06–1.50, *p* = 0.010) and cholelithiasis (OR 2.06, 95% CI: 1.04–4.08, *p* = 0.038) in patients without DM [33], while our study found a neutral effect regarding these side effects when comparing tirzepatide to placebo in individuals without DM. Specifically, the RR for gallbladder disorders was 1.26 (95% CI: 0.70–2.27, *p* = 0.43), and for cholelithiasis, it was 1.18 (95% CI: 0.69–2.03, *p* = 0.55) (Figure S7A,B). Additionally, our analysis indicated that the risk of acute pancreatitis was also neutral with tirzepatide compared with placebo in this patient population (RR 1.51, 95% CI: 0.42–5.38, *p* = 0.52) (Figure S7C).

Interestingly, a real‐world observational study comparing semaglutide and tirzepatide found that the risks of GI side effects, including gastroparesis, gastroenteritis, bowel obstruction, cholecystitis, cholelithiasis, and pancreatitis, were similar between the two medications [34]. However, a majority of patients in that study had type 2 DM, differing from our study cohort, which included those without DM. To definitively determine if there is a difference in these side effects between the two drugs in patients without DM, an RCT is needed. The ongoing SURMOUNT‐5 trial (NCT05822830), which is comparing tirzepatide and semaglutide in patients with overweight or obesity but without DM, is expected to provide answers to these questions [35].

Among the non‐GI side effects, notable ones include injection site reactions (OR 14.65, 95% CI: 5.81–31.70) and alopecia (OR 5.76, 95% CI: 2.95–11.23) (Figure S8B,C). The increased incidence of injection site reactions may be attributed to the medication's formulation, which could potentially affect patient adherence. Alopecia may be associated with significant weight loss experienced with tirzepatide, possibly leading to nutrient deficiencies.

The various side effects of tirzepatide, particularly GI, pose the risk of medication discontinuation. The study by Pan et al. indicated that in a mixed cohort of patients (both with and without type 2 DM), tirzepatide did not lead to a statistically significant increase in serious adverse events compared with placebo or other weight loss medications [12]. Similarly, Liu et al. found that tirzepatide did not significantly raise the risk of serious adverse events in patients without DM (OR 0.95, 95% CI: 0.69–1.30) [15]. However, that study noted a higher risk of treatment discontinuation due to adverse events in patients receiving tirzepatide versus placebo (OR 3.27, 95% CI: 3.40–5.33) [15]. Similar to the above studies, in our study, the risk of serious adverse events was not statistically significant (RR 0.93, 95% CI: 0.76–1.13) (Figure 6A). In addition, our study also showed that the RR for mortality was not statistically significant (RR 0.65, 95% CI: 0.28–1.51) (Figure 6B). However, we found a statistically significant RR for serious GI events (RR 3.07, 95% CI: 2.03–4.66) (Figure 6C). Furthermore, the percentage of participants discontinuing tirzepatide due to adverse events compared with placebo was also statistically significant, with an RR of 2.29 (95% CI: 1.74–3.01) (Figure 6D). These findings highlight the potential for serious GI side effects with tirzepatide, which may limit its acceptability in certain patient populations and necessitate close monitoring.

The SURMOUNT trials included in this meta‐analysis defined and adjudicated serious adverse events as those resulting in hospitalization, life‐threatening conditions, persistent or significant disability/incapacity, congenital anomalies or birth defects, death, or requiring medical intervention to prevent these outcomes. Serious GI adverse events were classified as those involving the GI system that met any of these criteria. Although the incidence of gallbladder disorders, cholelithiasis, and pancreatitis did not differ between tirzepatide and placebo, an increased risk of serious GI adverse events was observed. This difference may be attributed to other GI‐related adverse effects, such as nausea, vomiting, and diarrhea, which, depending on severity, could meet the criteria for serious events as defined in the trials.

The sustainability of weight loss following tirzepatide discontinuation remains a concern. While data on this topic are limited, the SURMOUNT‐4 trial provides valuable insights [10]. In this study, 36 weeks of tirzepatide treatment resulted in a mean weight reduction of 20.9%. However, discontinuing the medication led to a mean weight regain of 14.0% (95% CI: 12.8–15.2) after 1 year [10]. Further randomized controlled trials and real‐world studies are needed to better understand the long‐term effects of treatment discontinuation.

Our study has certain limitations. Although all the included studies employed a once‐weekly dosing regimen, they utilized varying dosages (Table 1). Additionally, the duration of the studies differed, with most lasting 52 weeks or more, while one was relatively short at just 18 weeks. These variations in dosage and study duration may have introduced heterogeneity into the pooled analysis. To mitigate this, we employed a random effects model when heterogeneity was detected. Despite these limitations, our study offers valuable insights into the diverse benefits and side effects of tirzepatide therapy for weight loss in individuals without DM.

In the field of weight loss, tirzepatide has emerged as a promising medication. Upcoming trials are expected to provide further insight into its benefits. Notably, the SURMOUNT‐5 trial (NCT05822830) is currently underway, comparing tirzepatide to semaglutide in patients with overweight or obesity but without type 2 DM [35]. Other upcoming trials are expected to provide details into the cardiovascular benefits associated with this treatment. These include the SUMMIT Trial (NCT04847557), which investigates tirzepatide in participants with heart failure with preserved ejection fraction (HFpEF) and obesity [36], and the SURPASS‐CVOT Trial (NCT04255433), which compares tirzepatide to dulaglutide concerning major cardiovascular events in individuals with type 2 DM [37]. While significant weight loss benefits are being observed with this medication, and future studies may clarify its cardiovascular outcomes, it is essential to remain mindful of the potential side effects and ensure that patients are well informed, emphasizing the need for close monitoring.

## 5 Conclusion

This study shows that in individuals with overweight or obesity without DM, once‐weekly subcutaneous tirzepatide can be beneficial with significant weight loss, albeit with an increased risk of GI side effects, serious GI adverse events, and medication discontinuation because of the side effects. However, the risk of overall serious adverse events is not elevated when compared with placebo.

## Author Contributions

Sharath Kommu (SK) contributed to the concept and design of the study, developed the search strategy, conducted the search and screening, extracted the data, and drafted and reviewed the manuscript. Param P Sharma (PS) reviewed the search results and the collected data, appraised the quality of evidence, and reviewed the manuscript. Rachel M Gabor (RG) assessed and verified the data, performed the statistical analysis, and reviewed the manuscript. All authors contributed to the critical revision of the manuscript, had full access to the data, and accept the responsibility to submit for publication.

## Conflicts of Interest

The authors declare no conflicts of interest.

## Supporting information


**Figure S1** Forest plots showing the change in BMI in kg/m2 (A) and waist circumference in cm (B) in patients with overweight or obesity without diabetes mellitus treated with tirzepatide versus placebo. BMI—body mass index, SD—standard deviation, MD—mean difference, CI—confidence interval, MTD—maximum tolerated dose, kg/m2 ‐kilograms per square meter, cm—centimeters.
**Figure S2:** Forest plots showing the change in HbA1C percentage (A) and fasting glucose level (mg/dL) (B) in patients with overweight or obesity without diabetes mellitus treated with tirzepatide versus placebo. HbA1C—glycated hemoglobin, SD—standard deviation, MD—mean difference, CI—confidence interval, MTD—maximum tolerated dose.
**Figure S3:** Forest plots showing the change in systolic (A) and diastolic (B) blood pressures (mm Hg) in patients with overweight or obesity without diabetes mellitus treated with tirzepatide versus placebo. SD—standard deviation, MD—mean difference, CI—confidence interval, MTD—maximum tolerated dose.
**Figure S4:** Forest plots showing the change in the lipid profile: total cholesterol (A), triglycerides (B), and LDL (C) in patients with overweight or obesity without diabetes mellitus treated with tirzepatide versus placebo LDL—low‐density lipoprotein, SD—standard deviation, MD—mean difference, CI—confidence interval, MTD—maximum tolerated dose.
**Figure S5:** Forest plots showing the change in the lipid profile: VLDL (A), free fatty acids (B), and HDL (C) in patients with overweight or obesity without diabetes mellitus treated with tirzepatide versus placebo VLDL—very low‐density lipoprotein, HDL—high‐density lipoprotein, SD—standard deviation, MD—mean difference, CI—confidence interval, MTD—maximum tolerated dose.
**Figure S6:** Forest plots showing the gastrointestinal side effects—dyspepsia (A) and eructation (B) in patients with overweight or obesity without diabetes mellitus treated with tirzepatide versus placebo. RR—relative risk, CI—confidence interval, MTD—maximum tolerated dose.
**Figure S7:** Forest plots showing additional gastrointestinal side effects—gallbladder disorders (A), cholelithiasis (B), and pancreatitis (C) in patients with overweight or obesity without diabetes mellitus treated with tirzepatide versus placebo. RR—relative risk, CI—confidence interval, MTD—maximum tolerated dose.
**Figure S8:** Forest plots showing side effects—hypoglycemia (A), injection site reaction (B), and alopecia (C) in patients with overweight or obesity without diabetes mellitus treated with tirzepatide versus placebo. RR—relative risk, CI—confidence interval, MTD—maximum tolerated dose.
