# Supplementary material for: Efficacy and Safety of Tirzepatide on Weight Loss in Patients Without Diabetes Mellitus: A Systematic Review and Meta‐Analysis of Randomized Controlled Trials
Source: Obes Rev. 2025 Jun 13;26(11):e13961. doi: 10.1111/obr.13961 (PMC12531717; doi:10.1111/obr.13961)
Supplement: Supplementary file 1 — Figure S1 Forest plots showing the change in BMI in kg/m2 (A) and waist circumference in cm (B) in patients with overweight or obesity without diabetes mellitus treated with tirzepatide versus placebo. BMI—body mass index, SD—standard deviation, MD—mean difference, CI—confidence interval, MTD—maximum tolerated dose, kg/m2 ‐kilograms per square meter, cm—centimeters. Figure S2: Forest plots showing the change in HbA1C percentage (A) and fasting glucose level (mg/dL) (B) in patients with overweight or obesity without diabetes mellitus treated with tirzepatide versus placebo. HbA1C—glycated hemoglobin, SD—standard deviation, MD—mean difference, CI—confidence interval, MTD—maximum tolerated dose. Figure S3: Forest plots showing the change in systolic (A) and diastolic (B) blood pressures (mm Hg) in patients with overweight or obesity without diabetes mellitus treated with tirzepatide versus placebo. SD—standard deviation, MD—mean difference, CI—confidence interval, MTD—maximum tolerated dose. Figure S4: Forest plots showing the change in the lipid profile: total cholesterol (A), triglycerides (B), and LDL (C) in patients with overweight or obesity without diabetes mellitus treated with tirzepatide versus placebo LDL—low‐density lipoprotein, SD—standard deviation, MD—mean difference, CI—confidence interval, MTD—maximum tolerated dose. Figure S5: Forest plots showing the change in the lipid profile: VLDL (A), free fatty acids (B), and HDL (C) in patients with overweight or obesity without diabetes mellitus treated with tirzepatide versus placebo VLDL—very low‐density lipoprotein, HDL—high‐density lipoprotein, SD—standard deviation, MD—mean difference, CI—confidence interval, MTD—maximum tolerated dose. Figure S6: Forest plots showing the gastrointestinal side effects—dyspepsia (A) and eructation (B) in patients with overweight or obesity without diabetes mellitus treated with tirzepatide versus placebo. RR—relative risk, CI—confidence interval, MTD—maximum tolerated dose. [file OBR-26-e13961-s001.pdf]

Efficacy and Safety of Tirzepatide on Weight Loss in Patients Without Diabetes Mellitus: A Systematic Review and Meta-Analysis of Randomized Controlled Trials

Authors:

1. Sharath Kommu
2. Param P. Sharma
3. Rachel M. Gabor

Authors' addresses and contact information:

Author 1 (Corresponding author):
Sharath Kommu, MD, FRCP, FACP, FHM

- Lead Hospitalist,
  Department of Hospital Medicine,
  Marshfield Clinic Health System,
  1700 W Stout St,
  Rice Lake, WI, USA – 54868
  Email: sharathkommu@gmail.com

Author 2:
Param P. Sharma, MD, FACC, FHRS

- Cardiac Electrophysiologist,
  Department of Cardiology,
  Marshfield Clinic Health System,
  Marshfield, WI, USA – 54449
  Email: sharma.param@marshfieldclinic.org

Author 3:
Rachel M. Gabor, MS
Biostatistician,
Office of Research Computing and Analytics,
Marshfield Clinic Research Institute,
Marshfield, WI, USA – 54449.
Email: gabor.rachel@marshfieldresearch.org

Supplementary Tables

Table S1: Search strategies

Databases	Search strategies	Number of reports
PubMed	(tirzepatide) AND (((obesity) OR (overweight) OR (("without diabetes mellitus") OR ("without type 2 diabetes") OR (nondiabetic) OR (non-diabetic))) NOT (("with type 2 diabetes") OR ("with diabetes")) Filters: in the last 5 years	229
ClinicalTrials.gov	Search term: Tirzepatide (completed studies only)	38

Supplementary Figures

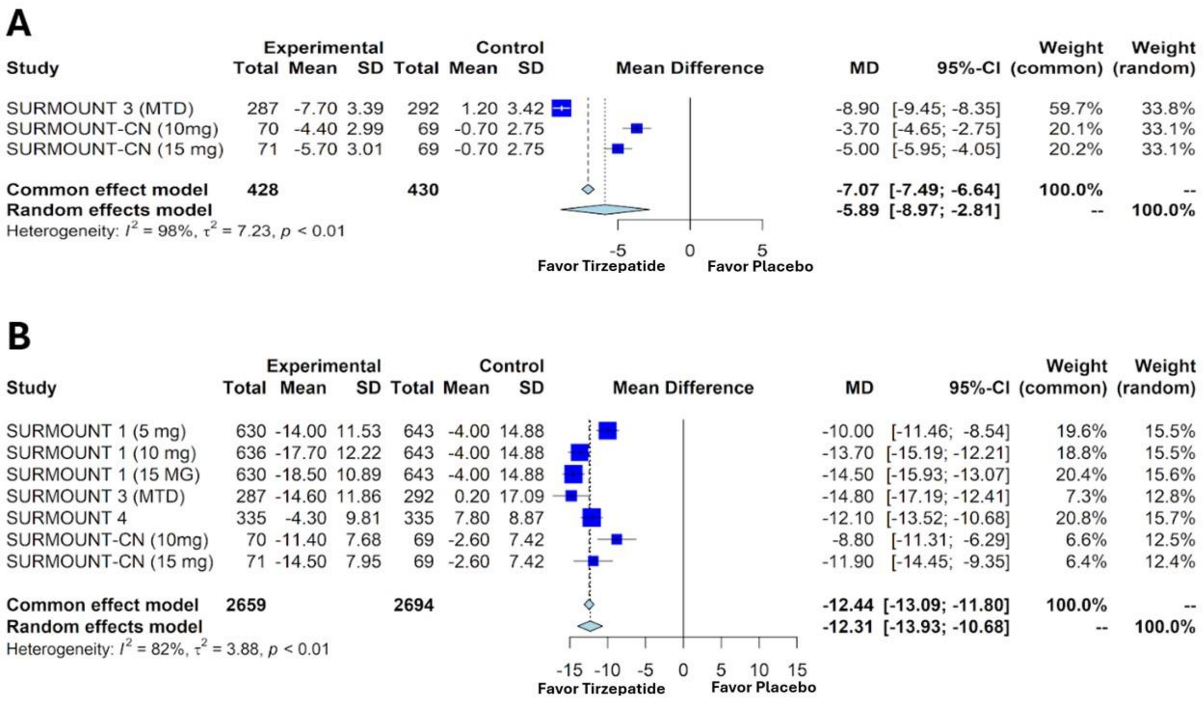

Figure S1: Forest plots showing the change in BMI in kg/m^2 (A) and waist circumference in cm (B) in patients with overweight or obesity without diabetes mellitus treated with tirzepatide versus placebo.
BMI—body mass index, SD - standard deviation, MD - mean difference, CI - confidence interval, MTD - maximum tolerated dose, kg/m2 -kilograms per square meter, cm - centimeters.

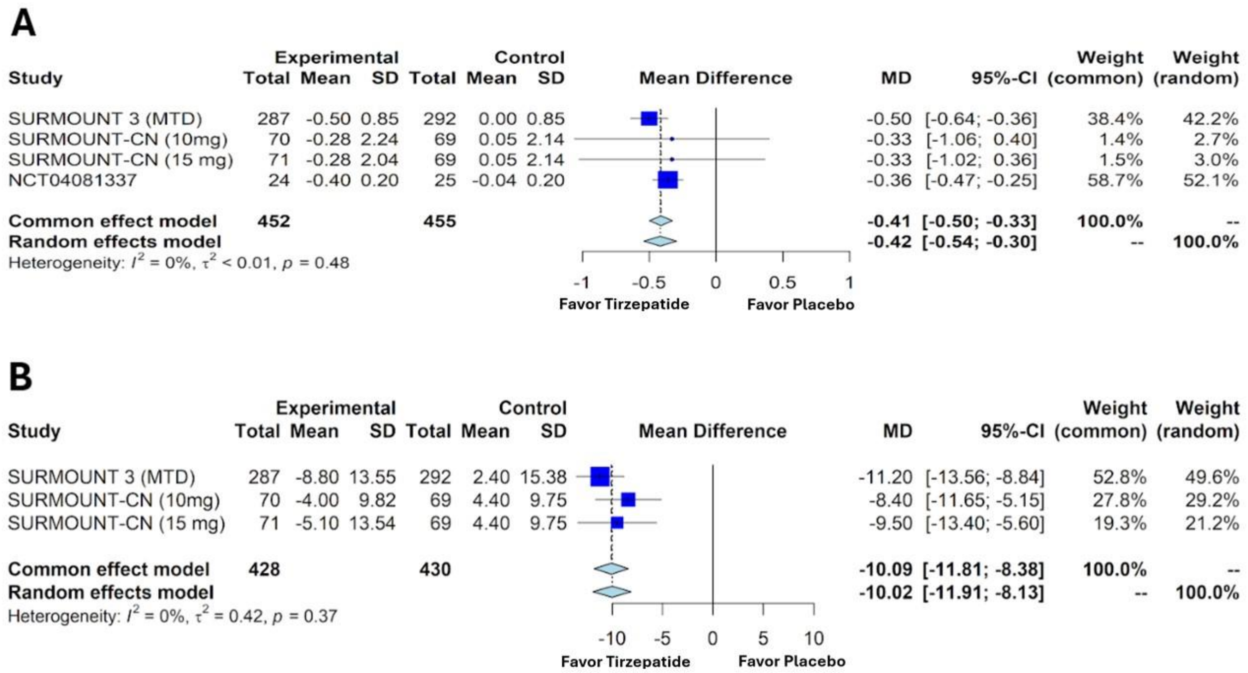

Figure S2: Forest plots showing the change in HbA1C percentage (A) and fasting glucose level (mg/dL) (B) in patients with overweight or obesity without diabetes mellitus treated with tirzepatide versus placebo. HbA1C - glycated hemoglobin, SD - standard deviation, MD - mean difference, CI - confidence interval, MTD - maximum tolerated dose.

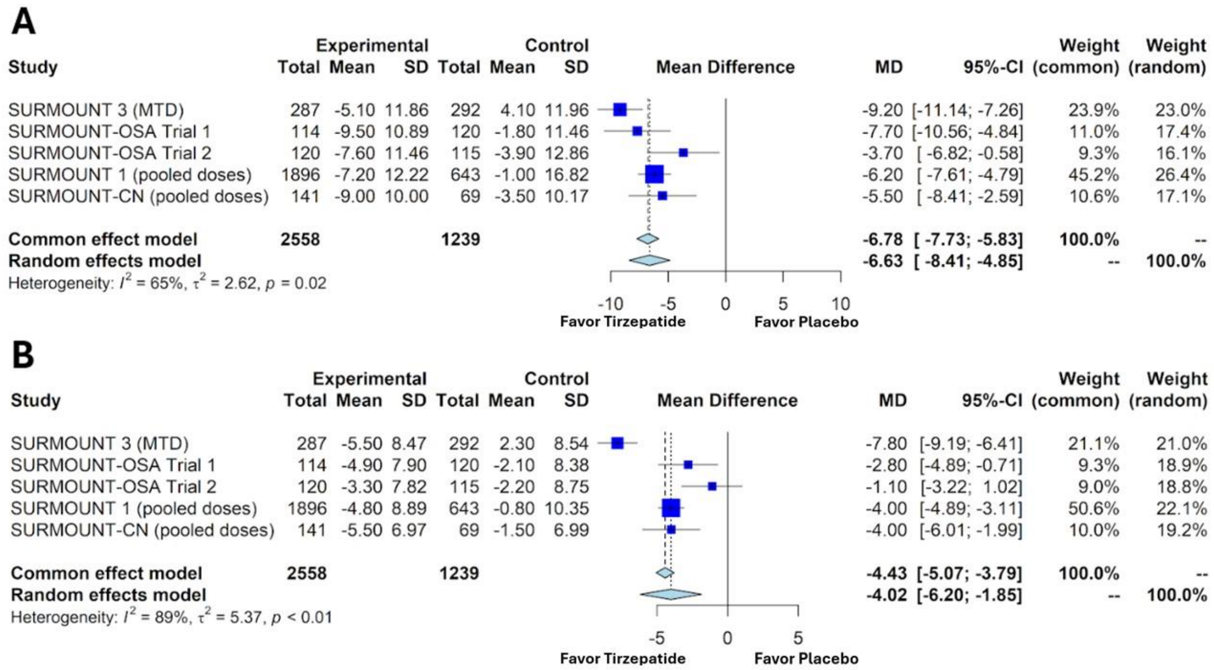

Figure S3: Forest plots showing the change in systolic (A) and diastolic (B) blood pressures (mm Hg) in patients

with overweight or obesity without diabetes mellitus treated with tirzepatide versus placebo. SD - standard deviation, MD - mean difference, CI - confidence interval, MTD - maximum tolerated dose.

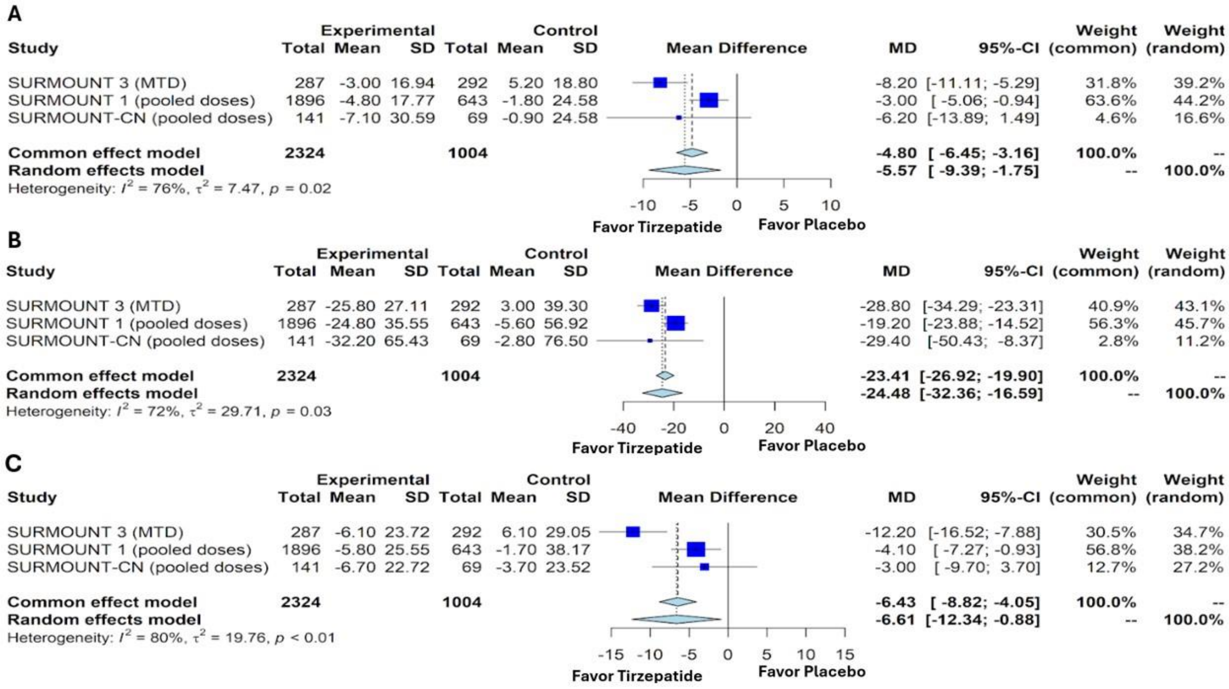

Figure S4: Forest plots showing the change in the lipid profile: total cholesterol (A), triglycerides (B), and LDL (C) in patients with overweight or obesity without diabetes mellitus treated with tirzepatide versus placebo LDL – low-density lipoprotein, SD - standard deviation, MD – mean difference, CI – confidence interval, MTD – maximum tolerated dose.

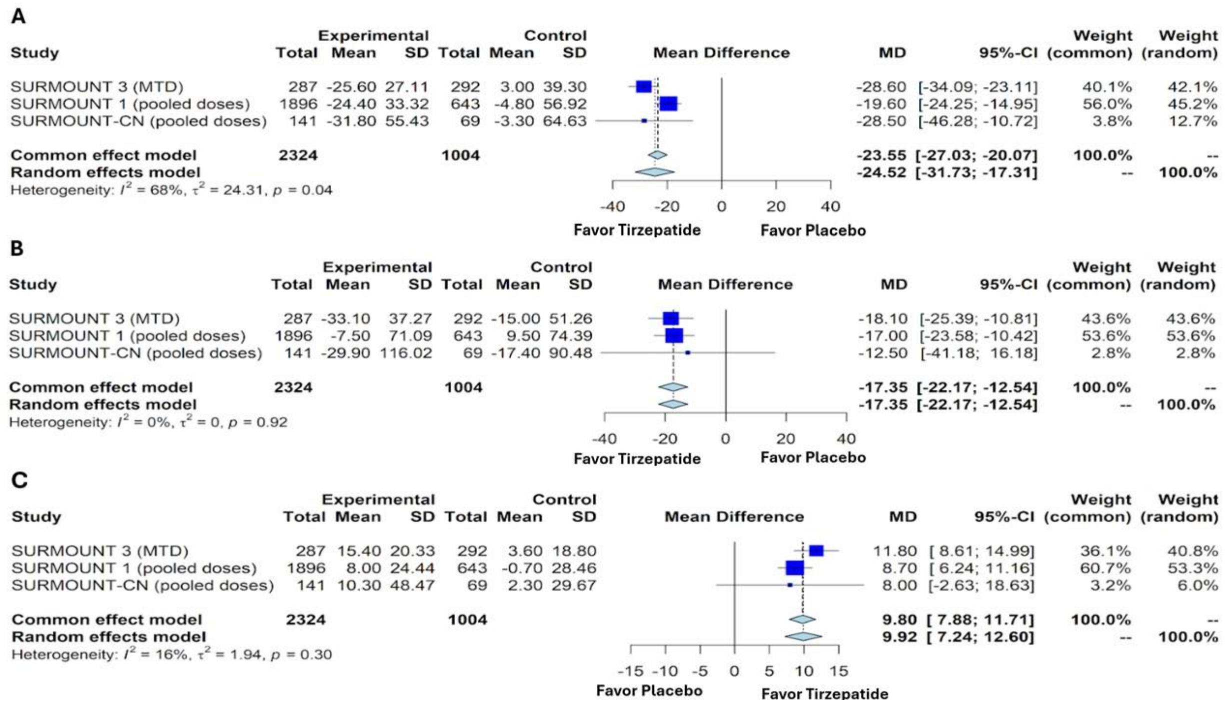

<u>Figure S5:</u> Forest plots showing the change in the lipid profile: VLDL (A), free fatty acids (B), and HDL (C) in patients with overweight or obesity without diabetes mellitus treated with tirzepatide versus placebo VLDL – very low-density lipoprotein, HDL – high-density lipoprotein, SD - standard deviation, MD – mean difference, CI – confidence interval, MTD – maximum tolerated dose.

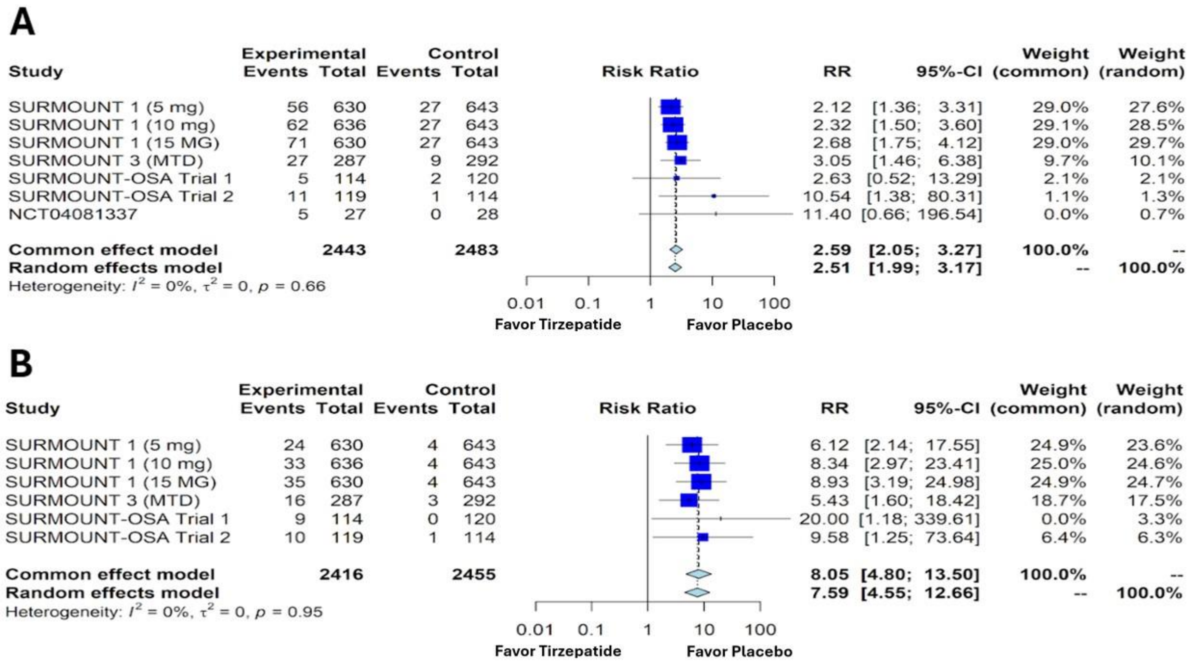

<u>Figure S6:</u> Forest plots showing the gastrointestinal side effects—dyspepsia (A) and eructation (B) in patients with overweight or obesity without diabetes mellitus treated with tirzepatide versus placebo. RR—relative risk, CI—confidence interval, MTD – maximum tolerated dose.

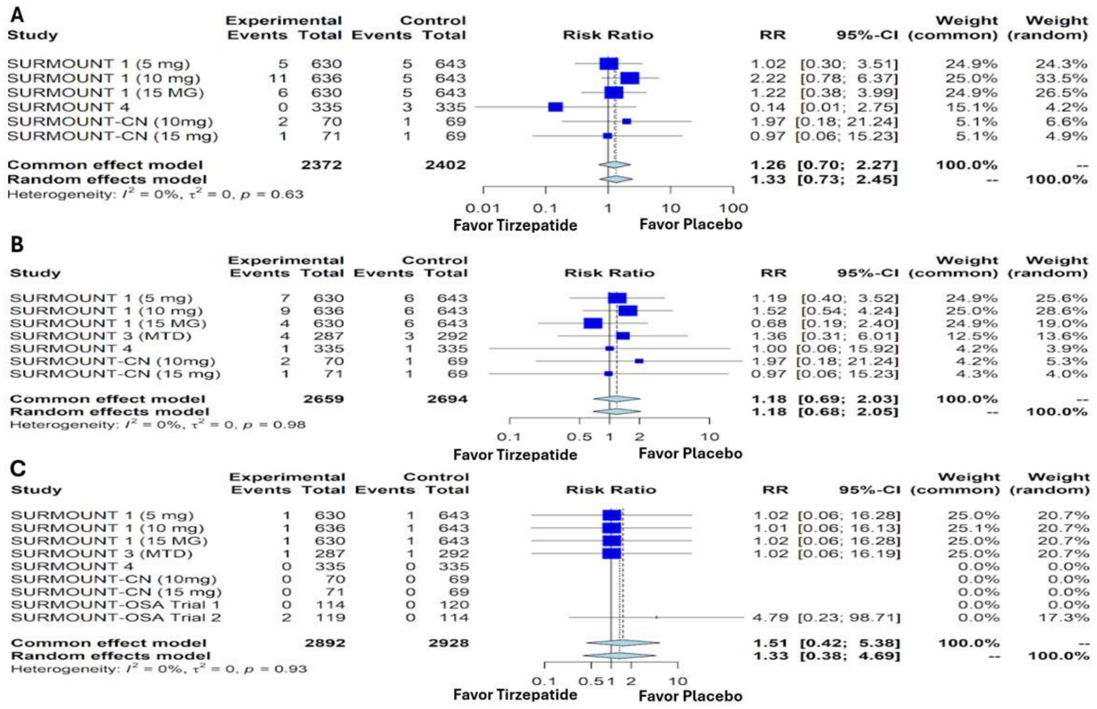

Figure S7: Forest plots showing additional gastrointestinal side effects—gallbladder disorders (A), cholelithiasis (B), and pancreatitis (C) in patients with overweight or obesity without diabetes mellitus treated with tirzepatide versus placebo. RR – relative risk, CI - confidence interval, MTD – maximum tolerated dose.

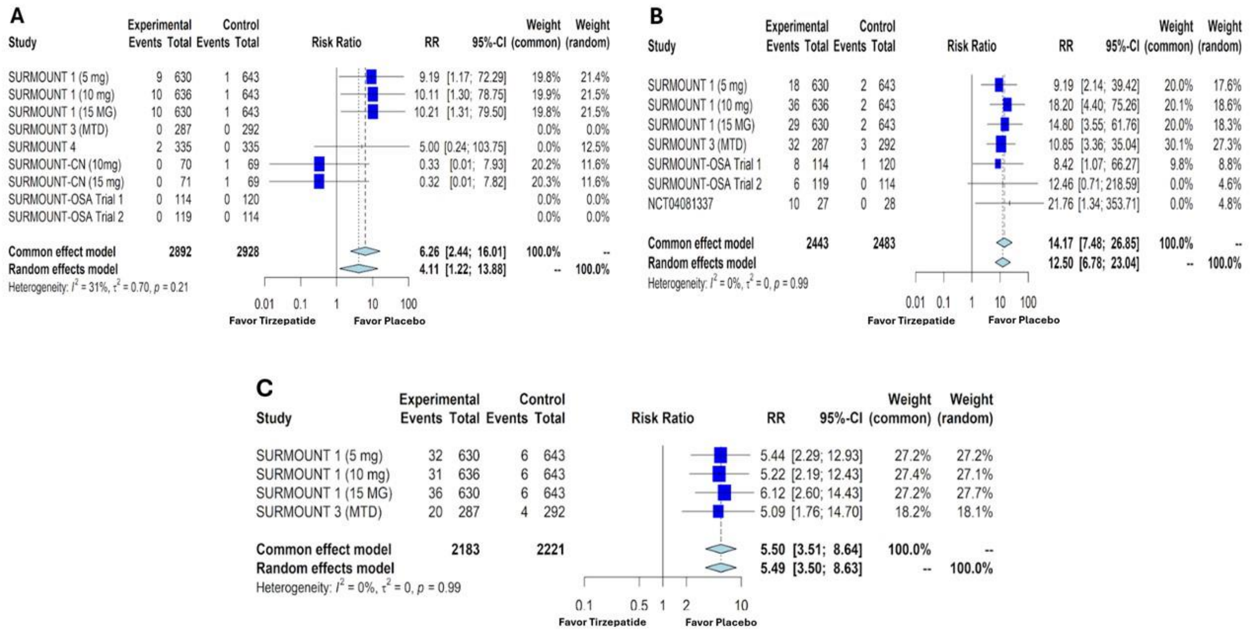

Figure S8: Forest plots showing side effects — hypoglycemia (A), injection site reaction (B), and alopecia (C) in patients with overweight or obesity without diabetes mellitus treated with tirzepatide versus placebo. RR—relative risk, CI—confidence interval, MTD – maximum tolerated dose.
